# Identification of Immune-Related Cells and Genes in Tumor Microenvironment of Clear Cell Renal Cell Carcinoma

**DOI:** 10.3389/fonc.2020.01770

**Published:** 2020-09-02

**Authors:** Bowen Du, Yulin Zhou, Xiaoming Yi, Tangliang Zhao, Chaopeng Tang, Tianyi Shen, Kai Zhou, Huixian Wei, Song Xu, Jie Dong, Le Qu, Haowei He, Wenquan Zhou

**Affiliations:** Department of Urology, Jinling Hospital, Medical School, Nanjing University, Nanjing, China

**Keywords:** ccRCC, tumor microenvironment, immunotherapy, TCGA, hub gene

## Abstract

Clear cell renal cell carcinoma (ccRCC) is one of the most common tumors in the urinary system. Progression in immunotherapy has provided novel options for the ccRCC treatment. However, the understanding of the ccRCC microenvironment and the potential therapeutic targets in the microenvironment is still unclear. Here, we analyzed the gene expression profile of ccRCC tumors from the Cancer Genome Atlas (TCGA) and calculated the abundance ratios of immune cells for each sample. Then, seven types of immune cells were found to be correlated to overall survival, and 3863 immune-related genes were identified by analyzing differentially expressed genes. We also found that the function of immune-related genes was mainly focused on ligand-receptor binding and signaling pathway transductions. Additionally, we identified 13 hub genes by analyzing the protein-protein interaction network, and seven of them are related to overall survival. Our study not only expands the understanding of fundamental biological features of microenvironment but also provides potential therapeutic targets.

## Introduction

Kidney cancer is one of the most common cancer in the world (1). Renal cell carcinoma (RCC) accounts for 85–90% of kidney cancer, while clear cell renal cell carcinoma (ccRCC) accounts for about 70–75% of RCC (2–6). ccRCC is frequently characterized by the von Hippel Lindau (VHL) tumor suppressor gene lose or inactivation, which leads to the overexpression of the hypoxia-inducible factor-2α (HIF2α), vascular endothelial growth factor (VEGF), and their downstream kinases (7–9). Multiple tyrosine kinase inhibitors (TKIs) have been developed as novel therapies (10), but the 5-year overall survival rate for advanced ccRCC remains under 30% (11, 12). Thus, it is necessary to develop novel therapies for ccRCC treatment.

The tumor microenvironment has been increasingly studied in the field of cancer immunotherapy in recent years. The tumor microenvironment is the non-cancerous cells and molecules surrounding the tumor cells, which consist of immune cells, blood vessels, adipocytes, mesenchymal stem cells, cancer-associated fibroblasts, and extracellular matrix (13, 14). The tumor microenvironment significantly influences therapeutic response and clinical outcomes through multiple signaling pathways (15). Thus, a better understanding of tumor microenvironment may help us develop novel therapies for ccRCC treatments.

To explore the immune microenvironment component in the ccRCC tumor and its interaction with the tumor cells, we first screened the survival-related immune cells in the ccRCC microenvironment. Then, we identified the immune-cell-specific genes by analyzing differentially expressed genes. To study the biological function of the immune-specific genes, we also enriched those genes in the Kyoto Encyclopedia of Genes and Genomes (KEGG), and Reactome pathways. Finally, we identified 13 hub genes by studying protein-protein interaction (PPI) networks and found seven genes correlated to overall survival. Our study not only revealed the biological features of the ccRCC microenvironment but also provided a novel view of ccRCC therapies.

## Materials and Methods

### Samples and Data Process

We selected 530 ccRCC patients from the Cancer Genome Atlas (TCGA) dataset and excluded 11 patients owing to the incomplete clinical data. The raw count data were downloaded from the TCGA website^1^. The transcripts per million (TPM) values were calculated with R software as the mRNA level of each gene. The clinical data were downloaded from the cbioportal website^2^ and summarized in Table 1.

**TABLE 1:** Patient clinical characteristics of The Cancer Genome Atlas cohort.

	Case (No.)	Percent (%)
Gender		
Male	337	64.9
Female	182	35.1
Age		
Median	60	
Range	26–90	
Fuhrman grade		
G1	14	2.7
G2	225	43.4
G3	206	39.7
G4	74	14.3
Stage		
I	261	50.3
II	54	10.4
III	122	23.5
IV	82	15.8
T Stage		
pT1	267	51.4
pT2	66	12.7
pT3	175	33.7
pT4	11	2.1

### Identification of Survival-Related Immune Cells

The abundance ratios of 22 types of immune cells were calculated with CIBERSORTx (16). The matrix of TPM values for all samples was uploaded to the CIBERSORTx website^3^ as the Mixture file. The LM22 matrix within the software was selected as the Signature gene file. The software was run in relative mode. Batch correction and quantile normalization were not performed in this run. The permutations for significance analysis were set to 100. Then, the Kaplan–Meier analysis was performed to identify the survival-related immune cells with the Survival R package based on the abundance ratio of each cell type. The Log-Rank test was used to analyze the survival data, and the medians of the abundance ratios for cell types were as the cut-off values. The relationship between the abundance ratios of immune cells and the pathological grades and the clinical stages were analyzed with the one-way ANOVA test.

### Identification of Immune-Related Genes

We identified the specific genes for each type of the survival-related immune cells by calculating the differentially expressed genes between the high- and low-immuno-infiltrated samples with the median of abundance ratios as the cut-off value. The differentially expressed genes were analyzed with the DEseq2 R package. The genes with | log_2_(foldchange)| > 1 and *p*-value < 0.05 were considered as specific genes for that cell type. The results were visualized with the UpSetR R package.

### Enrichment Analysis of Immune-Related Genes

The KEGG enrichment analysis was performed with the enrichKEGG function in the Clusterprofiler R package. The list of gene IDs was used as the input file. The Benjamini-Hochberg method was used to adjust the *p*-values. The cut-off of *p*-values was set to 0.05. The Reactome enrichment analysis was performed with the enrichPathway function in the ReactomePA R packages. The list of gene IDs was used as the input file. The parameters were set as follow: pAdjustMethod = “BH,” pvalueCutoff = 0.05, minGSSize = 10, and maxGSSize = 500. The enrichment results were visualized with the ggplot2 R package.

### Constriction of PPI Network and Identification of Hub Genes

The 933 immune-related protein-coding genes were imported into the STRING database^4^. The results with combined scores over 0.7 were kept and visualized with the Cytoscape software. To identify the hub genes, we clustered the genes within the PPI network with the MCODE plugin of the Cytoscape software using the following parameter: Degree Cutoff = 4, Node Score Cutoff = 0.3, K-core = 2, and Max. Depth = 100. The clusters containing over 40 proteins were used to extract hub genes. The hub genes were obtained with the CytoHubba plugin of the Cytoscape software.

### Relationship Between Immune Infiltration and Hub Genes

The Pearson correlation between the hub genes and all the 22 types of immune cells was calculated, and the result was visualized with the ggplot2 R package. Also, the correlation between the hub genes and the overall survival was calculated with the Log-Rank test.

## Results

### Identifying Survival-Related Immune Cells

**FIGURE 1:**
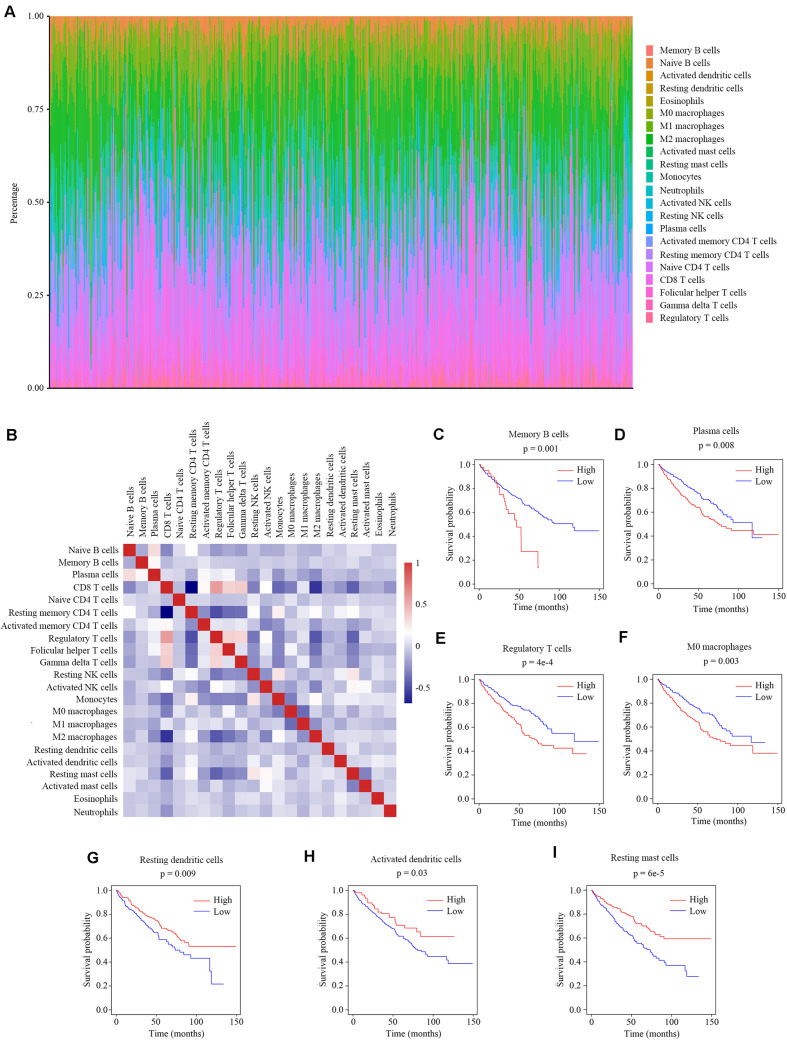
The relationship between the abundance ratios of immune cells and overall survival. **(A)** The abundance ratios of immune cells in the ccRCC samples. Each column represents a sample, and the column height indicates the abundance ratios of the certain immune cells in that sample. **(B)** The correlation coefficient between the abundance ratios of distinct immune cells. **(C–I)** The survival analysis for the abundance ratios of memory B cells, plasma cells, regulatory T cells, M0 macrophages, resting dendritic cells, activated dendritic cells, and resting mast cells.

Previous studies have reported the immune infiltration in the ccRCC tumors (17–20). Thus we explored the microenvironment components of ccRCC tumors by calculating the abundance ratios of 22 types of immune cells with the online software CIBERSORTx (Figure 1A). We found the T cells accounted for the most proportion (42.2 ± 14.2%), followed by the macrophages (33.7 ± 11.7%). We also found that the abundance ratios of some types of immune cells were correlated with each other (Figure 1B).

The abundance ratios of memory B cells, plasma cells, regulatory T cells, M0 macrophages, resting dendritic cells, activated dendritic cells, and resting mast cells were significantly correlated with the overall survival of ccRCC patients (Figures 1C–I). The higher abundance ratios of memory B cells, plasma cells, regulatory T cells, and M0 macrophages identified patients with worse prognosis, while the higher abundance ratios of resting dendritic cells, activated dendritic cells, and resting mast cells identified patients with better prognosis. These seven types of immune cells were considered as survival-related cells and analyzed in further study.

### Relationship Between Clinical Traits and Survival-Related Immune Cells

**FIGURE 2:**
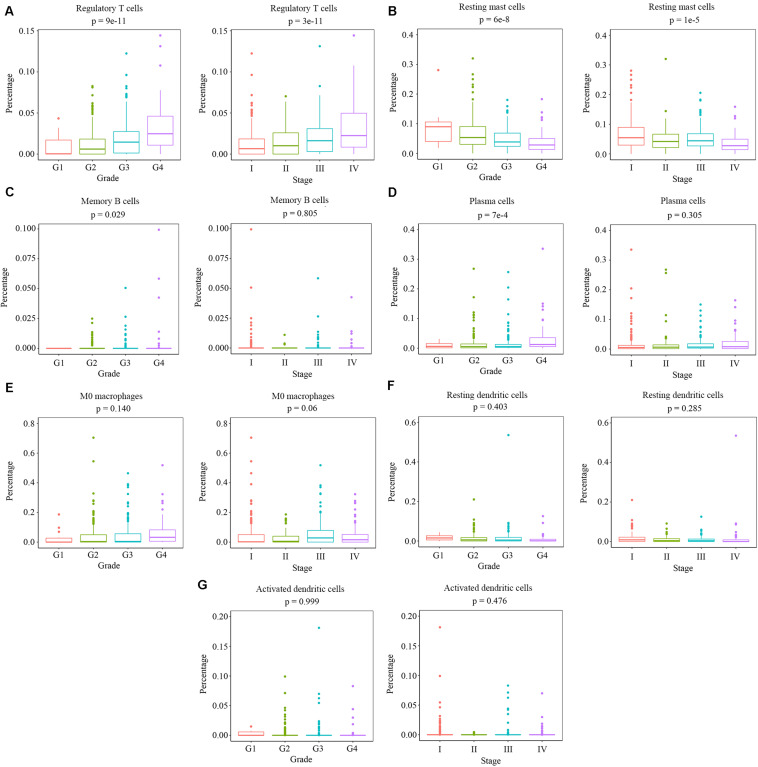
The relationship between the abundance ratios of the immune cells and clinical characteristics. **(A–G)** The abundance ratios of memory B cells, plasma cells, regulatory T cells, M0 macrophages, resting dendritic cells, activated dendritic cells, and resting mast cells in different pathological grades and clinical stages. Data are shown in boxplot format, and the dots represent the outliers.

We measured the relationship between clinical traits and the abundance ratios of survival-related immune cells. The abundance ratio of regulatory T cells increased with the increase of pathological grades and clinical stages (Figure 2A), while the abundance ratio of resting mast cells decreased with the increase of pathological grades and clinical stages (Figure 2B). The abundance ratio of memory B cells was statistically different in distinct pathological grades (*p* < 0.05). However, we still suggest that there was no change with the increase of pathological grades since the abundance ratio of memory B cells in most cases was relatively small and the difference probably attributed to the outliers (Figure 2C). The abundance ratio of plasma cells slightly increased in pathological grade 4 (Figure 2D). The abundance ratios of M0 macrophages, resting dendritic cells, and activated dendritic cells showed no significant difference in distinct grades or stages (Figures 2E–G). These results indicate that the abundance ratios of survival-related immune cells are not necessarily related to the pathological grade or clinical stage.

### Identification of Immune-Related Genes

**FIGURE 3:**
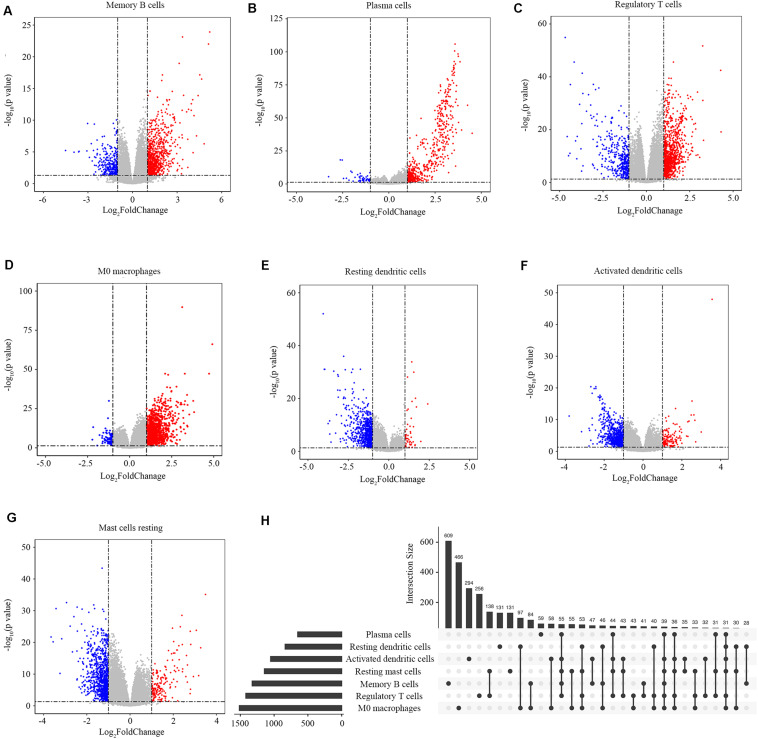
Identification of immune-related genes. **(A–G)** Gene expression profiles related to memory B cells, plasma cells, regulatory T cells, M0 macrophages, resting dendritic cells, activated dendritic cells, and resting mast cells. Data are presented with Volcano plots. The red/blue dots represent the upregulated/downregulated genes according to the criteria: | log_2_Foldchange| > 1 and adjusted *p*-value < 0.05. **(H)** The distribution of immune-related genes in the seven types of immune cells. Each black dot represents a set of genes that distributed in one type of immune cell. The numbers on the bar represent the counts of genes in this gene set. The dots connected with a black line represent a common set of genes distributed in more than one type of immune cell.

We screened the genes related to the abundance ratios of the survival-related immune cells with the method described in the Materials and Methods and found 3863 genes related to the abundance of the seven types of survival-related immune cells. In all these genes, 1325 genes were related to memory B cells, 651 to plasma cells, 1419 to regulatory T cells, 1515 to M0 macrophages, 837 to resting dendritic cells, 1052 to activated dendritic cells, and 1144 to resting mast cells (Figures 3A–G). The distribution of immune-related genes is shown in Figure 3H.

### Pathway Analysis of Immune-Related Genes

**FIGURE 4:**
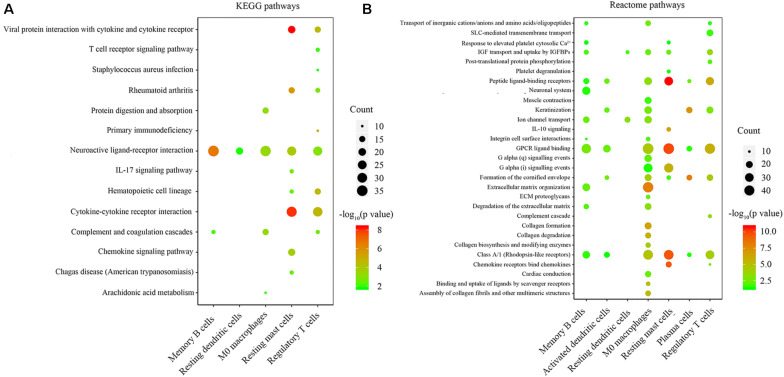
Enrichment analysis of genes related to immune cell infiltration. **(A,B)** the KEGG and Reactome pathway enrichment results of genes involved in each type of immune cells. The color indicates the significance of enrichment results, and the dot size indicates the count of genes enriched for each result.

We performed KEGG and Reactome pathway enrichment for each group of immune-related genes to explore the biological function of immune-related genes. The results are listed in Supplementary Tables S1, S2. The results with gene counts over ten are shown in Figure 4. The KEGG pathway enrichment results showed that the immune-related genes were mainly enriched in neuroactive ligand-receptor binding, cytokine-cytokine receptor interaction (Figure 4A). The Reactome pathways enrichment results showed that the immune-related genes were mainly enriched in G protein-coupled receptor (GPCR) ligand binding, peptide ligand-binding receptors (Figure 4B). These results indicate that the immune-related genes might be involved in ligand-receptor binding and signaling pathway transduction.

### Identification of Hub Genes

**FIGURE 5:**
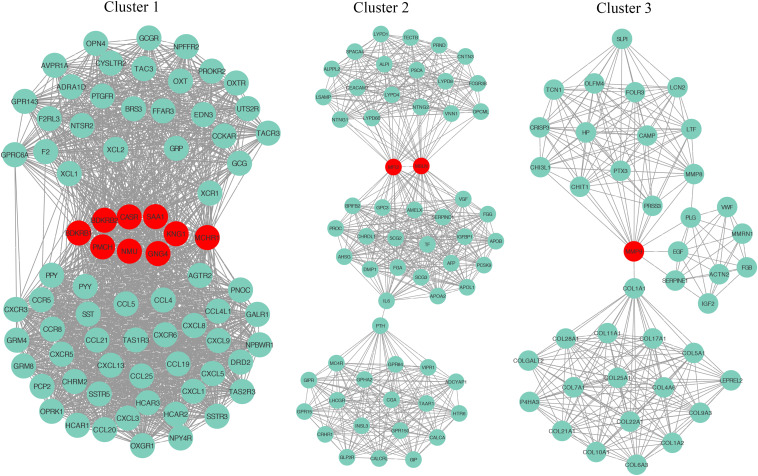
The identification of hub genes. Three gene clusters were identified from the PPI network by the MCODE plugin. The hub genes are highlighted in red dots.

To explore the detail of immune-related gene relationships, we constructed the PPI with all the protein-coding genes in the immune-related gene set. To identify the critical immune-related gene, we explored the gene clusters within the PPI network with the MCODE plugin of the Cytoscape software. Three clusters with no less than 40 genes were found and applied to identify the hub genes. Here, the Hub genes were those genes with the most interacted genes in the cluster. Finally, 13 genes were identified as the hub genes (Figure 5 and Table 2).

**TABLE 2:** List of the 13 hub genes.

Gene	Full name
CASR	Calcium sensing receptor
BDKRB1	Bradykinin receptor B1
MMP9	Matrix metallopeptidase 9
MSLN	Mesothelin
COL1A1	Collagen type I alpha 1 chain
NMU	Neuromedin U
KNG1	Kininogen 1
MCHR1	melanin concentrating hormone receptor 1
MFI2	Melanotransferrin 2
GNG4	G protein subunit gamma 4
BDKRB2	Bradykinin receptor B2
SAA1	Serum amyloid A1
PMCH	Pro-melanin concentrating hormone

### Relationship Between Clinical Traits and Hub Genes

**FIGURE 6:**
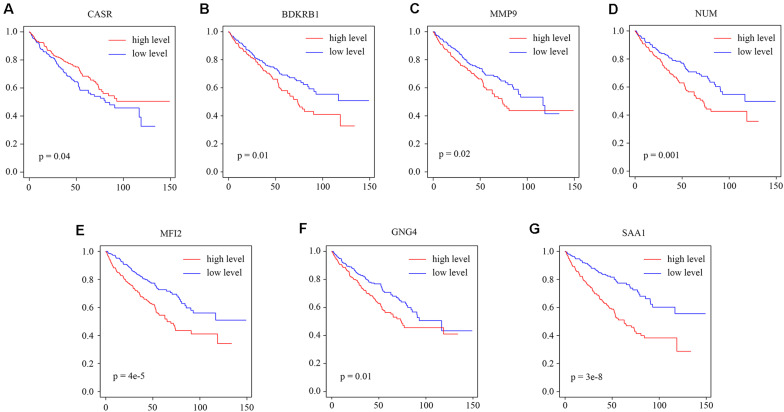
The relationship between the hub genes and overall survival. **(A–G)** The survival analysis for the hub gene CASR, BDKRB1, MMP9, NMU, MFI2, GNG4, and SAA1.

**FIGURE 7:**
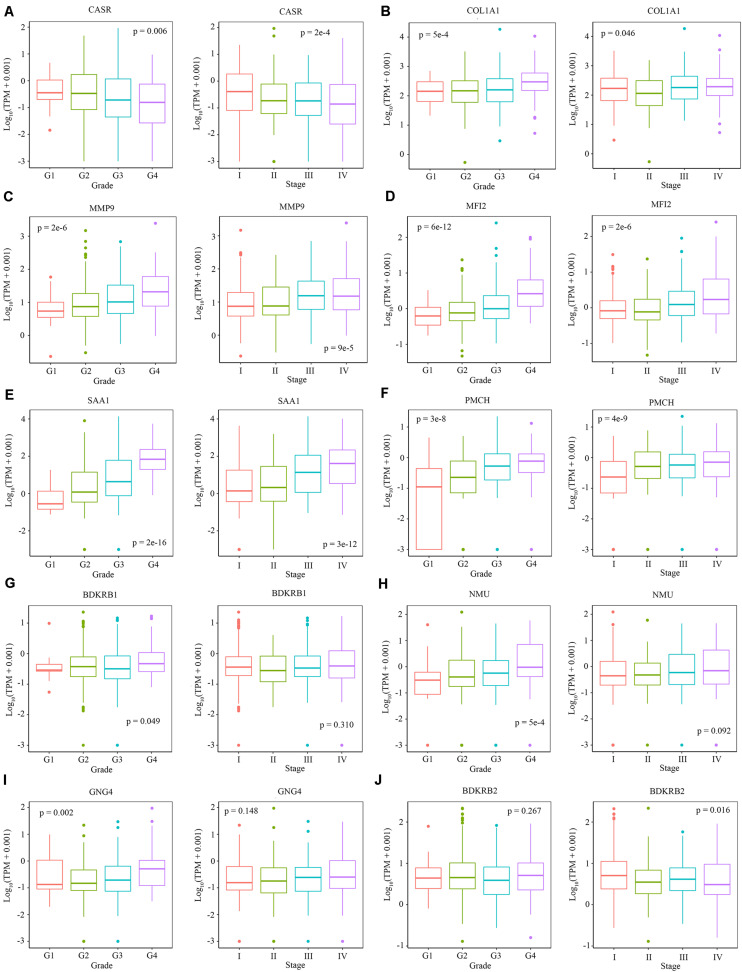
The relationship between the level of hub genes and clinical characteristics. **(A–J)** The level of CARS, COL1A1, MMP9, MFI2, SAA1, PMCH, BDKRB1, NMU, GNG4, and BDKRB2 in different pathological grades and clinical stages. Data are shown in boxplot format, and the dots represent the outliers.

We explored the relationship between the overall survival and the hub genes. We found that 7 out of 13 hub genes (CASR, BDKRB1, MMP9, NMU, MFI2, GNG4, and SAA1) are correlated to overall survival (Figure 6). We also explored the relationship between the clinical traits and the hub genes. The level of CASR decreased with the increase of pathological grades and clinical stages (Figure 7A). The level of COL1A1 increased in pathological grade 4 but decreased in clinical stage II (Figure 7B). Meanwhile, the levels of MMP9, MFI2, SAA1, and PMCH increased with the increase of pathological grades and clinical stages (Figures 7C–F). The levels of BDKRB1, NMU, and GNG4 increased only with the increase of pathological grades (Figures 7G–I). The level of BDKRB2 decreased with the increase of clinical stages (Figure 7J). The levels of MSLN, KNG1, and MCHR1 showed no difference in distinct pathological grades or clinical stages (Supplementary Figure S1).

**FIGURE 8:**
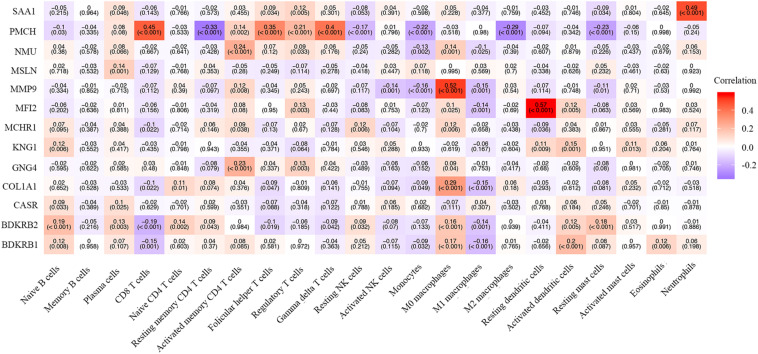
The correlation between the hub genes and immune cells. The Pearson correlation coefficients are presented with a heatmap. The number without brackets is the Pearson correlation coefficients, and the number with brackets is the *p*-value.

We also explored the correlation between hub genes and the abundance ratios of 22 types of immune cells. We found that multiple hub genes were correlated to the abundance ratio of certain types of immune cells (Figure 8). For instance, the level of MMP9 was positively correlated to the abundance ratio of M0 macrophages, while the level of PMCH was negatively correlated to the abundance ratio of resting mast cells. These results indicate that the hub genes might play a vital role in the function of the immune cells.

## Discussion

As the comprehensive molecular characterization of ccRCC has been performed (3, 20–23), genetic and epigenetic prognostic markers have been widely studied (24, 25). Since immune cell infiltration has been widely reported in ccRCC (3, 17–20), the prognostic value of the immune-based markers has emerged. Prognostic models based on tumor-associated immune cells and genes have been developed (26–28). In this study, we pursued to expand the range of immune prognostic tools by exploring microenvironment component of ccRCC. We analyzed the ccRCC microenvironment by calculating the abundance ratios of 22 types of immune cells. The results showed a variation of the abundance ratios among distinct patients, which not only supported those previous research but also indicated that the ccRCC microenvironment might be complex.

We found that seven types of survival-related immune cells, including memory B cells, plasma cells, regulatory T cells, M0 macrophages, resting dendritic cells, activated dendritic cells, and resting mast cells, were correlated with overall survival. A previous study has identified ICOS + Treg cells as a prognostic marker in localized ccRCC, which is consistent with our results (29). Also, Eckl et al. analyzed immune cell infiltration of 41 ccRCC samples with flow cytometry and found that patients with a high level of NK cell infiltration had better cancer-specific survival (30). This result is consistent with our results that the higher abundance ratios of resting dendritic cells and activated dendritic cells identify patients with better prognosis. These research, to a certain extent, indicate that these seven types of immune cells might be predictors for ccRCC prognosis.

The survival-related immune cells in the microenvironment may be the potential therapeutic targets. Several studies demonstrated that macrophages constitute up to 50% of a tumor mass, forming a major component of the tumor microenvironment (31, 32). A widely accepted theory on macrophage subtypes is the plastic modal that macrophages can be activated into classically polarized tumor-suppressive M1 and alternatively polarized tumor-promoting M2 subtypes. M2 macrophages promote ccRCC progression due to their immune-suppressive property (33, 34). Several therapeutic agents targeting macrophages have been developed in recent years (35, 36). According to our data, the abundance ratio of macrophages is over 30% in ccRCC tumors. Therefore, macrophages might be a potential target for ccRCC treatment.

We also screened the immune-related genes and analyzed their biological functions. Most of the immune-related genes were enriched in neuroactive ligand-receptor binding, cytokine-cytokine receptor interaction, GPCR ligand binding, peptide ligand-binding receptors. These results imply that the immune-related genes may be associated with ligand-receptor binding and its downstream signaling pathways that may be potential therapeutic targets. For instance, G protein-coupled receptor 68 (GPR68), a proton-sensing GPCR, plays a vital role in multiple types of tumors (37, 38). Additionally, several immune-related genes were enriched in the interleukin (IL)-17 signaling pathway. IL-17 is mainly produced by Th17 cells, a subtype of T helper cells (39). The activation of IL-17 signaling pathways leads to the overexpression of Chemokines, Cytokines and matrix metallopeptidases (MMPs) through multiple signaling pathways (40).

We also identified 13 hub genes from the immune-related genes and found seven of them (CASR, BDKRB1, MMP9, NMU, MFI2, GNG4, and SAA1) are correlated with overall survival. CASR, a Calcium-sensing receptor, is expressed in the immune cells including macrophages, eosinophils, and monocytes (41–43). Several studies have reported that CASR expression can be induced by multiple cytokines (44, 45). In murine macrophages, the CASR activates the NACHT, LRR, and NLRP3 inflammasome in a cAMP-dependent manner (46). Additionally, MMP9 is a downstream matrix metallopeptidase of the IL-17 signaling pathway. MMP9 promotes metastasis by degrading the extracellular matrix. Ma et al. reported that the level of MMP9 is higher in metastatic ccRCC than in primary ccRCC (47). The level of MMP9 is associated with poor prognosis in ccRCC patients (48). These results indicate that the hub genes may play a key role in the network of immune-related genes.

There are still limitations in our study. First, the analysis of immune-cell infiltration is based on the TCGA dataset and needs to be validated with samples from other sources. Second, the hub genes are identified from PPI networks based on the String database, which needs to be proved by experiment on cell line models. Third, the abundance ratio of memory B cells is zero in most cases, which means the conclusion on memory B cells relays on outliers. Although these results are statistically reliable, we should be cautious with the conclusion on memory B cells. Fourth, intra-tumor heterogeneity of pathological grades in ccRCC has been reported (49). Thus, we need to be cautious about the conclusion on the relation between pathological grades and immune-related cells and genes.

In the past decade, immune checkpoint inhibition therapy has been developed. Nivolumab, a monoclonal antibody targeting programmed death-1 (PD-1), was approved as the second-line treatment for advanced RCC in 2015. Mikami et al. explored the level of PD-1 and programmed death ligand 1 (PD-1L) in tumor-infiltrating immune cells in the tumor microenvironment of untreated and VEGF-TKI-treated primary ccRCC tissues. They found that the high level of PD-1 and PD-L1 in tumor-infiltrating immune cells was associated with the poor prognosis and the clinical response to VEGF-TKI treatment for metastatic ccRCC (50). These results indicate the potential effect of microenvironment on the immune checkpoint inhibition therapy. In conclusion, we identified seven types of survival-related immune cells and 13 hub genes, and seven of these genes were correlated to overall survival in ccRCC patients. These cells and genes can be considered predictors for prognosis, or as therapeutic targets for ccRCC. Our research not only provides a critical understanding of ccRCC microenvironments but also identifies the potential therapeutic targets.

## Data Availability Statement

Publicly available datasets were analyzed in this study. This data can be found here: The Cancer Genome Atlas (https://portal.gdc.cancer.gov/).

## Author Contributions

BD, SX, JD, LQ, HH, and WZ: study concept and design. BD, XY, and TZ: analysis and interpretation of data. BD and YZ: drafting of the manuscript. SX, CT, TS, KZ, and HH: critical revision of the manuscript. All authors contributed to the article and approved the submitted version.

## Conflict of Interest

The authors declare that the research was conducted in the absence of any commercial or financial relationships that could be construed as a potential conflict of interest.

## Footnotes

1 https://portal.gdc.cancer.gov

2 http://www.cbioportal.org

3 https://cibersortx.stanford.edu

4 https://string-db.org

## Supplementary Material

FIGURE S1 The relationship between the level of hub genes and clinical characteristics. **(A–C)** The level of MSLN, KNG1 and MCHR1 in different pathological grades and clinical stages. Data are shown in boxplot format, and the dots represent the outliers.
