# Supplementary figures and images for: Identification of Immune-Related Cells and Genes in Tumor Microenvironment of Clear Cell Renal Cell Carcinoma
Source: Front Oncol. 2020 Sep 2;10:1770. doi: 10.3389/fonc.2020.01770 (PMC7493752; doi:10.3389/fonc.2020.01770)

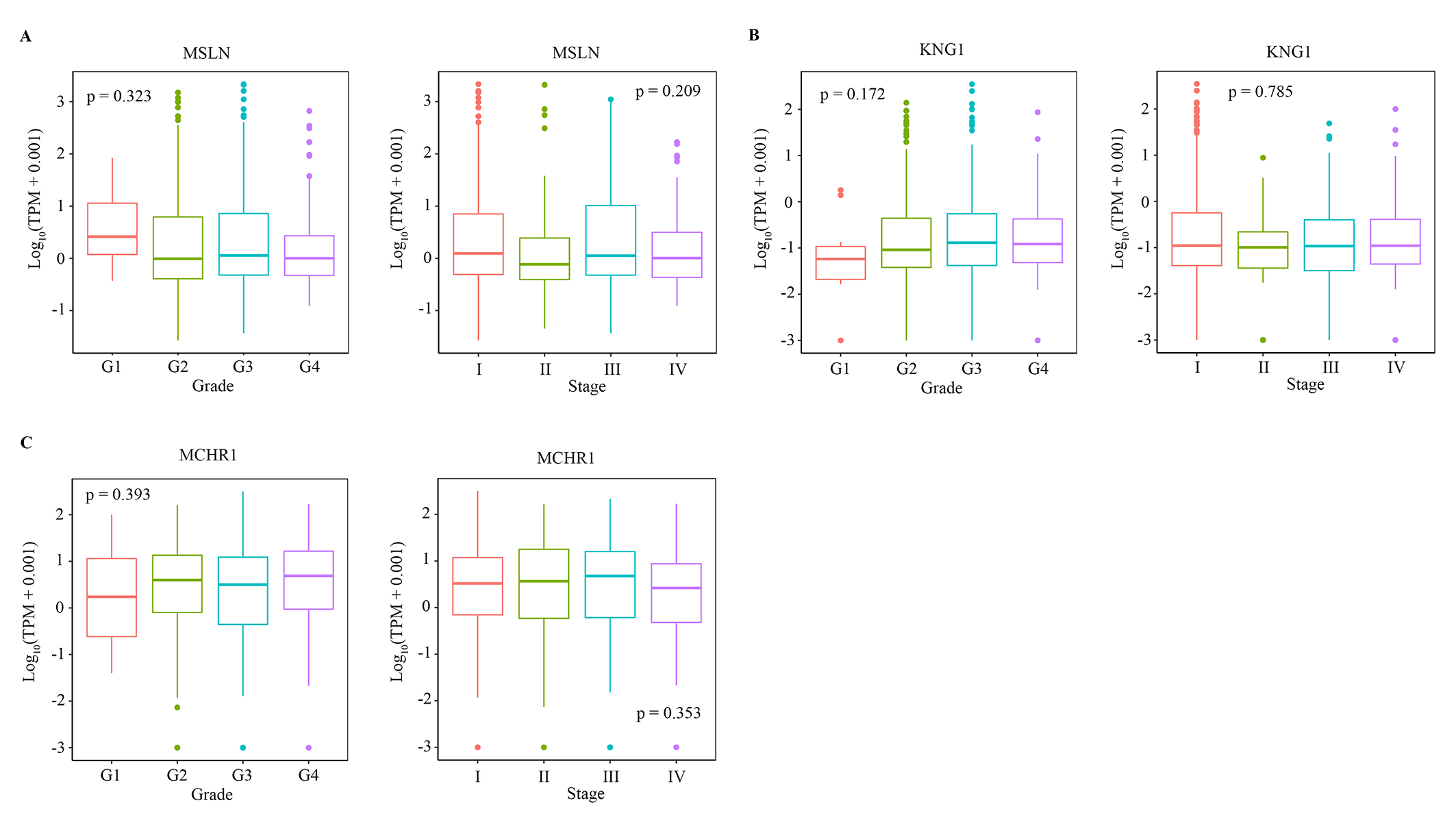

Supplement: FIGURE S1 — The relationship between the level of hub genes and clinical characteristics. (A–C) The level of MSLN, KNG1 and MCHR1 in different pathological grades and clinical stages. Data are shown in boxplot format, and the dots represent the outliers. [file Image_1.TIF]
